# Short versus Longer Implants in Sites without the Need for Bone Augmentation: A Systematic Review and Meta-Analysis of Randomized Controlled Trials

**DOI:** 10.3390/ma15093138

**Published:** 2022-04-26

**Authors:** Luigi Guida, Eriberto Bressan, Gennaro Cecoro, Armando Davide Volpe, Massimo Del Fabbro, Marco Annunziata

**Affiliations:** 1 Multidisciplinary Department of Medical-Surgical and Dental Specialties, University of Campania “Luigi Vanvitelli”, 80138 Naples, Italy; luigi.guida@unicampania.it (L.G.); armandovolpe1396@gmail.com (A.D.V.); marco.annunziata@unicampania.it (M.A.); 2 Department of Neurosciences, School of Dentistry, University of Padova, Via Giustiniani 2, 35100 Padova, Italy; eriberto@studiobressan.com; 3 Department of Biomedical, Surgical and Dental Sciences, University of Milan, 20122 Milan, Italy; massimo.delfabbro@unimi.it; 4 IRCCS Galeazzi Orthopedic Institute, 20161 Milan, Italy

**Keywords:** short implants, non-atrophic sites, implant survival rate, systematic review, meta-analysis

## Abstract

Objectives: The present systematic review and meta-analysis aims to analyse the clinical performance of short compared to longer implants inserted in sites without the need for bone augmentation. Methods: The protocol of the present PRISMA-driven meta-analysis was registered on PROSPERO (CRD42021264781). Electronic and manual searches were performed up to January 2022. All Randomized Controlled Trials (RCTs) comparing short (≤6 mm) to longer (≥8.5 mm) implants placed in non-atrophic and non-augmented sites were included. The quality of the included studies was assessed using the Cochrane risk of bias tool for randomized clinical trials (RoB 2) and the quality of evidence was determined with the Grading of Recommendations Assessment, Development, and Evaluation (GRADE) approach. A meta-analysis was performed on implant survival rate, marginal bone level change (MBLc), and technical and biological complications at the available follow-up time points. The power of the meta-analytic findings was determined by trial sequential analysis (TSA). Results: From 1485 initial records, 13 articles were finally included. No significant difference was found in the survival rate between short and long implant at any follow-up (moderate quality of evidence). Significantly more bone loss for long implants at 1 and 5 years from implant placement and more technical complications with short implants at 10 years were found. No other significant inter-group differences in terms of MBLc and biological complications were detected. Conclusions: Moderate evidence exists suggesting that short implants perform as well as longer ones in the rehabilitation of edentulous sites without the need for bone augmentation. Further long-term, well-designed RCTs, however, are still needed to provide specific evidence-based clinical recommendations for an extended use of short implants in non-atrophic sites.

## 1. Introduction

The rehabilitation of edentulous patients with dental implants constitutes a stable and highly predictable acquisition of modern dentistry, corroborated by well-documented clinical results and long survival [1,2]. The presence of an adequate bone volume at the edentulous site still represents a basic requirement for dental implants to be correctly placed. Dimensional reduction of the alveolar process, however, usually happens after tooth extraction, and adjunctive loss of bone may be caused by trauma, periodontal disease, or atrophy [3]. In these cases, additional reconstructive surgeries, e.g., vertical bone augmentation procedures or sinus lift, may be applied to allow standard-length implant placement, although they invariably associate with relevant limitations, such as longer treatment time, additional costs, increased postoperative morbidity, and a higher risk of complications [4,5,6,7,8].

A widely documented alternative to augmentation surgical procedures is represented by the rehabilitation of atrophic edentulous sites with implants of reduced length, which have reached a considerable diffusion in the last few years with very promising clinical results [9,10,11,12].

Several advantages are linked to the use of short implants, including ease of handling, reduced surgical invasiveness, and a low risk of injuring noble anatomical structures, thus sustaining the concept of a “stress minimizing surgery” [9]. Short implants, however, are not free from risks and complications, due to the higher crown-implant ratio and to the lower bone-to-implant contact area with respect to longer fixtures [13].

The clinical performance of short implants has been widely investigated in the recent literature, with not univocal findings. Higher failure rates have been historically associated with short implants compared to longer ones [14,15]. More recent studies showed, for short implants, clinical outcomes comparable to, or even better than, longer implants placed in both native or augmented bone, as confirmed by several systematic reviews [8,12,16,17,18,19,20,21,22,23,24,25].

The reasons for such a great variability may be found in several elements of heterogeneity among studies.

The definition of “short dental implant”, for instance, greatly differs from <10 mm to ≤8 mm and, more recently, ≤7 mm [15,26,27,28]. In this sense, in the 2015 EAO consensus on the use of short implants for dental rehabilitation [11], the authors recommend, for future research, to identify the minimal length for a predictable survival of implants (redefining the concept itself of “short” implant) and to perform clinical trials with an appropriate design to reliably compare these therapeutic concepts in the long term.

Furthermore, one should consider that the greatest part of the existing studies are designed to compare short implants placed in pristine atrophic bone with long implants inserted in augmented sites. Furthermore, short- and medium-term data (1 to 5 years from loading) are prevalent, and longer follow-ups are lacking.

In the 2018 ITI Consensus Conference [12], the authors highlight, in the recommendation for future research, the need for RCTs comparing short and longer implants in pristine bone sites without the need for vertical bone augmentation.

To reliably compare short and long implants, the same clinical scenario should be guaranteed to both groups: similar bone quality and height (which implies the placement of short implants in sites able to receive longer ones), as well as same prosthetic design to rehabilitate the same type of edentulism.

Such strictly designed comparisons would provide, on one hand, further support to the efficacy of short implants in their routine use in case of atrophic bone, and, on the other hand, could also help to extend their clinical indications to their use also in case of non-atrophic sites, instead of long implants.

The aim of the present systematic review and meta-analysis is to analyse the available scientific evidence from randomized controlled trials (RCTs) regarding the clinical performance of short (≤6 mm) compared to longer (≥8.5 mm) implants exclusively placed in sites without the need for bone augmentation.

## 2. Materials and Methods

### 2.1. Study Registration

The review protocol was registered with the PROSPERO International Prospective Register of Systematic Reviews with the identification number CRD42021264781.

### 2.2. Reporting Format

The 27-item Preferred Reporting Items for Systematic Reviews and Meta-Analyses (PRISMA) statement [29] was followed for the summary and description of the search process results.

Patient, Intervention, Comparison, Outcome, Time (PICOT) question

1. The focused question was formulated following the PICOT format [30], where:
2. Patients (P): Patients receiving fixed rehabilitations supported by implants placed in sites without bone augmentation procedures in the mandible and/or the maxilla.
3. Intervention (I): dental implants with length ≤ 6 mm.
4. Comparison (C): dental implants with length ≥ 8.5 mm.
5. Outcome (O): implant survival rate as primary outcome, marginal bone level change, and biological and technical complication rate as secondary outcomes.
6. Time (T): follow-up ≥ 1 year from prosthetic loading.

### 2.3. Focused Question

Do short (≤6 mm) implants perform as well as longer dental implants (≥8.5 mm) placed in sites without the need for bone augmentation?

### 2.4. Information Sources and Search Strategy

A computerized, systematic search of literature was performed using MEDLINE (PubMed, www.ncbi.nlm.nih.gov/pubmed, (accessed on 17 January 2022)), EMBASE, SCOPUS, clinicaltrial.gov, open grey until 17 January 2022. No date or language restriction was applied. The detailed search strategy for each electronic database consulted is presented in Table 1.

A manual search was performed on the major journals of implantology: Clinical Implant Dentistry and Related Research, Clinical Oral Implant Research, Clinical Oral Investigations, International Journal of Periodontics and Restorative Dentistry, Journal of Clinical Periodontology, Journal of Dental Research, Journal of Dentistry Journal of Periodontal Research, Journal of Periodontology.

Corresponding authors were contacted as needed to obtain information about missing data or unpublished material.

### 2.5. Outcome Variables

The primary outcome was considered the implant survival rate (SR), defined as the ratio between the number of implants still present at the follow-up and those originally randomized (intention to treat analysis), at implant level.

As secondary outcomes, the following outcomes were considered:

- Marginal bone level change (MBLc), defined as the difference in crestal bone height between baseline and follow-up measures, considered at patient or implant level.
- Technical complication rate, considered at patient level, concerning the number of any technical complication, such as prosthesis fracture, screw loosening or fracture, implant fracture, etc., occurred until the follow-up.
- Biological complication rate, considered at patient level, concerning the number of biological complications, i.e., peri-implant mucositis and peri-implantitis, occurred until the follow-up.

### 2.6. Eligibility Criteria

Studies were deemed eligible if they met the following inclusion criteria:

(a) RCTs comparing short (≤6 mm) implants in the test group and longer implants (≥8.5 mm) in the control group;
(b) studies with a follow-up period of at least 12 months from prosthetic loading;
(c) studies in which the implants were restored with a fixed prosthesis;
(d) studies where both test and control implants were placed exclusively in sites without the need for bone augmentation in the mandible and/or maxilla.

The following studies were excluded:

(a) preclinical in vitro or animal studies;
(b) case reports and case series;
(c) prospective and retrospective observational studies;
(d) non-randomized controlled trials;
(e) reviews and meta analysis;
(f) studies with insufficient information for any quantitative analysis.

### 2.7. Population Characteristics

In accordance with the study design, two patient groups were created: patients with short implants (≤6 mm) and longer dental implants (≥8.5 mm).

### 2.8. Study Selection and Data Extraction

All articles were initially screened by two independent reviewers based on titles and abstracts and imported to a reference manager to remove duplicates. Afterwards, full texts were carefully examined and included or excluded using a predetermined data extraction form based on the aforementioned eligibility criteria. Any disagreement was resolved via discussion between the two reviewers. The level of agreement between the reviewers regarding study inclusion was calculated using kappa coefficient. Data concerning patient and treatment characteristics, as well as clinical outcomes for each available follow-up, were independently extracted from all the eligible studies by two reviewers. When more articles referred to the same study, data and information reported were compared and, if possible, integrated. In the case of conflicting data among articles referring to the same study, only one of them was chosen. When additional information was required, authors were contacted. If no or inconclusive responses were obtained, data were excluded from the analysis.

### 2.9. Meta-Analysis

A meta-analysis was performed on implant survival rate as primary outcome and MBLc and technical and biological complications as secondary outcomes. A separate analysis was performed on the baseline chosen for MBLc measurements (prosthetic loading or implant placement). Following the principles of “intention-to-treat” analysis, the total number of initially randomized patients (or implants) for each included study was considered for calculation of the overall effect size of implant survival rate and complications. When data at implant and patient level were both reported in the same study, patient-level data were preferred. MBLc data were separately analysed from both implant placement and prosthetic loading as baseline; MBLc data provided by studies performing immediate loading were included in both the analyses. Negative and positive MBLc values were used to indicate bone loss and bone gain, respectively. Effect sizes were displayed as mean difference (MD) or risk ratio (RR) for continuous and dichotomous variables, respectively, with 95% confidence intervals. Forest plots were created to illustrate the effects of the different studies and global estimation.

RevMan 5 software (Review Manager, version 5.4, the Cochrane Collaboration, 2020, London, UK) was used to perform the statistical analyses. Statistical significance was defined as a *p*-value < 0.05. The study-specific estimates were pooled with the random-effects models if heterogeneity across tested trials with the Chi^2^ (Cochran Q) test (*p* < 0.1) and I^2^ statistics > 50% proved to be high [31]. If the meta-analysis contained a sufficient number of trials to make a visual inspection of the plot meaningful (ten trials minimum), funnel plots were considered as a tool for assessment of publication bias.

### 2.10. Trial Sequential Analysis

Trial Sequential Analysis (TSA) was performed for the main outcome (implant survival), to evaluate the power of the meta-analysis and to adjust the results for type I and II errors. TSA 0.9.5.10 Beta software was used (Copenhagen Trial Unit Centre for Clinical Intervention Research Department, Copenhagen, Denmark). The fixed-effects model was selected for the meta-analysis. The required information size (RIS) and alpha-spending monitoring boundaries were estimated by setting type I and type II error at 5% and 20% (power of 80%), respectively. To calculate RIS, for both test (short implants) and control (long implants) arms, the incidence (positive events) was estimated according to the findings of the meta-analysis, without applying correction for heterogeneity. The corresponding graphics allowed to determine if the cumulative Z-curve (blue line) crosses the RIS threshold (vertical red line) and the trial sequential monitoring threshold (horizontal red line). If so, it was considered that the studies had an adequate sample size, and their results were valid. Otherwise, it was assumed that the available information was inadequate, and more evidence was needed.

### 2.11. Risk of Bias and Quality of Evidence

The quality of the included trials was assessed using the Cochrane Risk of Bias Tool for randomized clinical trials (RoB 2) (updated on 22 August 2019) [32] by two calibrated examiners independently to ensure agreement on the scoring system. Each study was judged to be at low, moderate (some concerns), or high risk of bias based on five domains: (1) bias arising from the randomization process; (2) bias due to deviations from intended interventions; (3) bias due to missing outcome data; (4) bias in measurement of the outcome; (5) bias in selection of the reported result. The overall risk of bias of each study was considered “low” when the risk of bias was judged low for all domains. It was judged to raise “some concerns” when at least one domain raised some concerns but no domain was at a high risk of bias. It was judged “high” when at least one domain was at high risk or the study was judged to have some concerns for multiple domains in a way that substantially lowers confidence in the result. The outcome assessed for risk of bias was the implant survival rate. The aim of the review team was to investigate the effect of assignment to the interventions at baseline, regardless of whether the interventions were received as intended (‘intention-to-treat effect’). Articles reporting results from the same study were grouped and evaluated together.

Following Grades of Recommendation, Assessment, Development and Evaluation (GRADE) methods [33], a ‘Summary of findings’ table for the primary outcome of the present meta-analysis (survival rate), including all follow-up periods of each comparison group, was developed by the GRADEpro GDT web application, http://gradepro.org, (accessed on 20 February 2020). The quality of the body of evidence was assessed by considering the overall risk of bias of the included trials, the directness of the evidence, the inconsistency of the results, the precision of the estimates, and the risk of publication bias. The quality of evidence was categorized as high, moderate, low, or very low.

## 3. Results

### 3.1. Study Selection

The electronic search retrieved a total of 1525 articles. 766 records were screened after duplicate removal and 18 remained after title and abstract evaluation. One additional article [34] was collected through manual screening and unpublished data of another article were obtained directly from authors [35]. After full-text assessment, another seven articles were excluded (Table 2).

**Figure 1:**
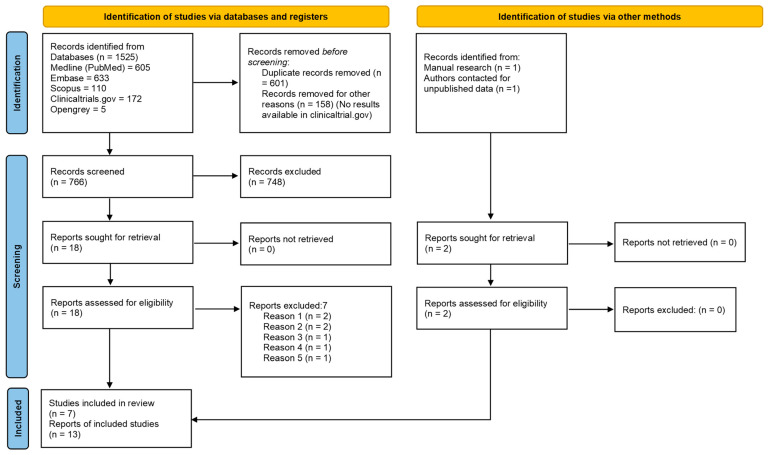
PRISMA 2020 flowchart of the selection process.

The *k* value for the inter-reviewer agreement for potentially pertinent papers was 0.865 (for the selection of titles and abstracts) and 0.894 (for the selection of full-text articles). Finally, data from 12 articles published between 2013 and 2021 [34,43,44,45,46,47,48,49,50,51,52] and one unpublished article [35] were included in the present systematic review. The selection process is shown in Figure 1.

### 3.2. Characteristics of the Included Articles

The characteristics of the 13 articles [34,35,43,44,45,46,47,48,49,50,51,52,53] included in this systematic review are summarized in Table 3.

Three articles [47,48,52] reported data pertaining to the same cohort at 1, 3, and 5 years of follow-up. Two articles followed the same patients at 1 and 3 years [34,51]. Four articles [35,43,44,49] followed 2 cohorts from 1 to 5 years and another 2 articles [46,53] at 5-year and 10-year follow-ups.

Ten articles [34,43,44,45,46,47,48,51,52,53] reported the outcomes from implants placed both in the upper and lower jaws, whereas 2 studies (3 articles) [35,49,50] considered only mandible implants. Two articles [43,44] reported the outcome of maxillary and mandibular implants separately. A parallel-group design was followed in all the studies but one [45], where some patients were allowed to receive both test and control implants.

The total number of inserted implants was 1066; 540 short implants were inserted (50.7%) with a minimum of 23 implants [50] and a maximum of 124 [34,51] implants; 526 long implants were inserted (49.3%) with a minimum of 23 [50] and a maximum of 116 implants [34,51].

The total number of treated patients was 454, and in one study [45] some patients received both short and long implants; 235 patients were treated with short implants (51.8%) with a minimum of 11 [46,53] and a maximum of 75 patients [34,51]; 234 patients were treated with long implants (51.5%) with a minimum of 13 [46,53] and a maximum of 75 patients [51,53].

Nine articles [35,45,46,47,48,49,50,52,53] considered short implants of 6 mm, 2 articles [43,44] of 5 mm, and 2 articles [34,51] of 4 mm. Two articles [34,51] considered control implants as ≥8.5 mm long; 4 articles as 10 mm long [45,46,50,53]; 5 articles as 11 mm long [35,47,48,49,52]; and 2 as ≥11.5 mm long [43,44].

The majority of the included studies focused on partial edentulism in the posterior jaws rehabilitated by single or 2–3 splinted crowns [34,45,46,47,48,50,51,52,53], while only 2 studies (4 articles) [35,43,44,49] focused on the rehabilitation of total edentulism by full-arch prostheses.

In terms of the loading protocol [54], in three articles (two studies), implants were immediately loaded [43,44,50], six articles (three studies) followed an early loading protocol (1–8 weeks) [45,46,47,48,52,53], and the remaining performed conventional loading (>8 weeks) [34,35,49,51].

In five articles (three studies) [34,43,44,50,51], post-extraction implants were also included, whereas all the other implants were exclusively placed in healed bone (at least 4 months after tooth extraction), although the exact surgical timing [55] was often not specified.

### 3.3. Risk/Confounding Factors

Factors such as smoking, implant surface, bone quality, primary stability, crown-to-implant ratio, periodontal status, and systemic diseases were screened to ascertain if they had been assessed and analysed in the originally included studies.

Six articles (3 studies) [34,35,43,44,49,51] included heavy smokers (≥10 cigarettes/day) and a similar inter-group distribution was reported. The other studies excluded or avoided the recruitment of heavy smokers [46,47,48,52,53]. In two studies [45,50], smokers were included but not categorized in heavy or light smokers.

All the studies included only systemically healthy patients or patients with controlled systemic diseases, or patients without general contraindications to implant surgery.

All of them evaluated the periodontal status of the patients and excluded patients with active periodontitis or reported that the periodontal treatment was performed if needed. Only one study (3 articles) [47,48,52] reports the number of patients with a history of periodontitis for each study group.

Only two studies (3 articles) assessed the quality of bone [43,44,45], showing no inter-group differences. The majority of them [34,45,47,48,50,51,52] systematically evaluated the primary stability of implants and, where available, no significant inter-group differences were reported. One study reported bone quality only for lost implants [48].

Three studies reported the anatomical crown-to-implant ratio [45,46,47], and 2 studies assessed the presence of patients with bruxism [45,47], but none of them provided information about intergroup-distribution, while severe bruxism was an exclusion criteria of other two studies (3 articles) [35,49,50].

### 3.4. Risk of Bias and Quality of Evidence

A total of 7 studies (13 articles) were evaluated.

**Figure 2:**
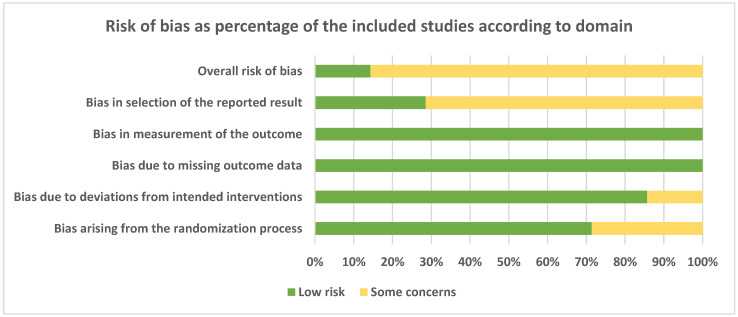
Risk of bias assessment expressed as a percentage of the included studies according to domain.

Risk of bias assessment expressed as percentage of the included studies according to domain is presented in Figure 2. Most of the domains were fulfilled by all the studies. For some of them, however, some concerns were expressed for possible biases arising from the randomization process (about 30% of the studies), deviation from intended interventions (about 15%), and in the selection of the reported results (about 70% of the studies).

According to the GRADE system, pooling of studies from 1 to 10 years of follow-up [34,35,44,45,48,50,53] provided moderate-quality evidence (Table 4) for a comparable survival rate between short and long implants.

### 3.5. Meta-Analysis

#### 3.5.1. Survival Rate

The overall survival rates of the reported implants for the short and long implants, respectively, were 96.85% and 98.48% at 1 year (7 articles), 96.14% and 98.76% at 3 years (4 articles), 95.40% and 98.44% at 5 years (5 articles), and 96.15% and 100% at 10 years (one article).

**Figure 3:**
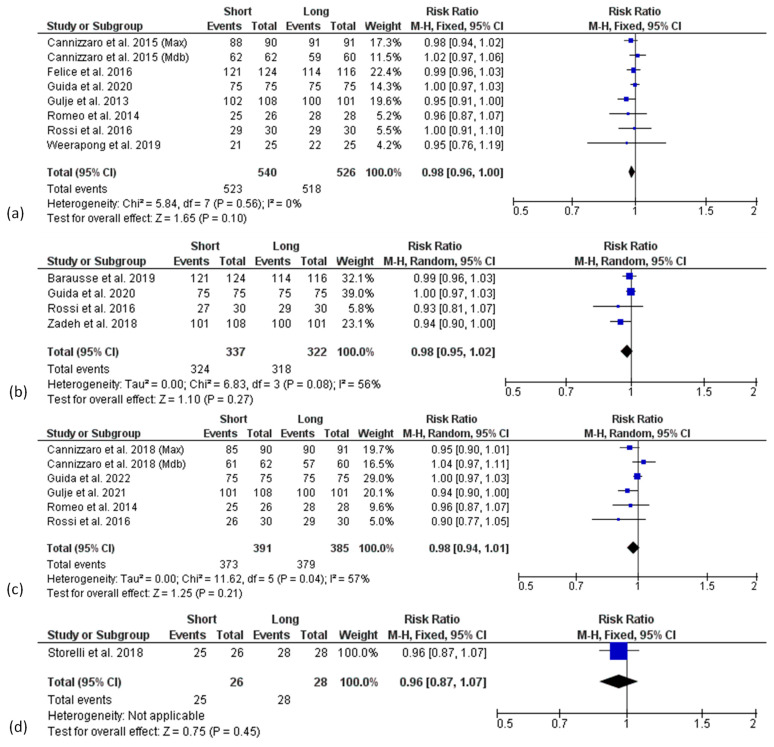
Forest plots (risk ratio, RR) of the survival rate comparing short and long implant groups in (**a**) 1-year, (**b**) 3-year, (**c**) 5-year and (**d**) 10-years results. RR < 1 indicated a survival rate of short implants lower than the long implants.

Forest plots of the survival rate (RR) comparing short and long implants at different follow-ups are shown in Figure 3. Mantel–Haenszel (MH)-weighted RR < 1 indicated a lower survival rate of short implants than the long implants. Data were analysed at implant level. The estimates were pooled using a random effect model for 3- and 5-years of follow-up analysis, due to the high heterogeneity found (I^2^ = 56%, *p* < 0.1 and I^2^ = 57%, *p* < 0.1, respectively), while for all the other analyses, a fixed-effect model was used.

The meta-analysis of 7 studies (8 data sets) at 1-year follow-up revealed no significant difference of survival rate between short and long implants with a risk ratio of 0.98 (95% CI: 0.96–1.00; *p* = 0.10) (Figure 3a).

A similar finding was observed in the analysis of 3-year results from 4 studies [34,45,49,52], with a risk ratio of 0.98 (95% CI: 0.95–1.02; *p* = 0.27) (Figure 3b), as well as in the analysis of 5-year results from 5 studies (6 data sets) [35,44,45,46,48] with a risk ratio of 0.98 (95% CI: 0.94–1.01; *p* = 0.21) (Figure 3c).

The 10-years follow-up analysis included only one study [53] and showed no significant difference between short and long implants in terms of survival rate with a risk ratio of 0.96 (95% CI: 0.87–1.07; *p* = 0.45) (Figure 3d).

Although not statistically significant, a trend for higher survival rates in the longer implant group at all follow-up times was found.

**Figure 4:**
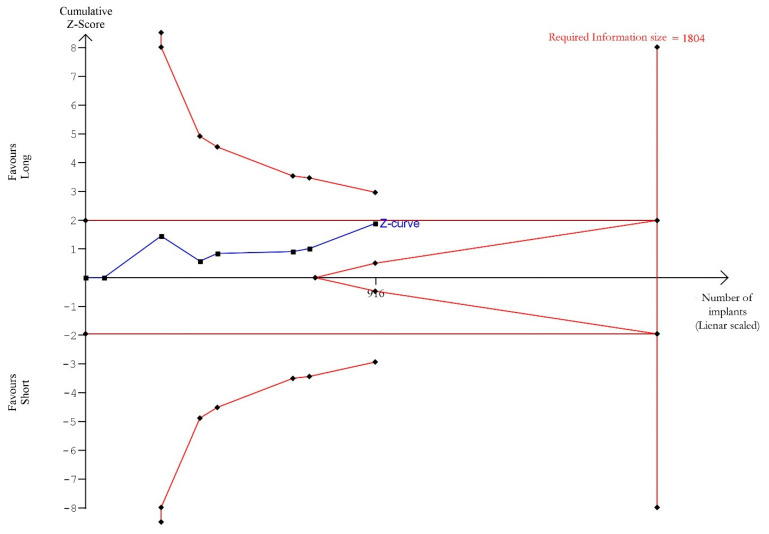
Trial Sequential Analysis (TSA) of implant survival rate comparing short with long implants (follow-up range 1 to 10 years). Two-sided graph. The cumulative Z-curve showing treatment effect (blue line) does not cross the trial sequential monitoring boundaries (horizontal red line), revealing a not significant effect. Additionally, the Z-curve does not surpass the required information size threshold (vertical red line), revealing weak power of evidence.

Trial Sequential analysis was carried out considering, for each study, only the results of the latest follow-up (from 1 to 10 years) [34,35,44,45,48,50,53]. TSA showed (Figure 4) a trend for better outcomes in favour of the long implants, without achieving significance. The cumulative Z-curve (blue line) kept below the trial sequential monitoring threshold (horizontal red line), revealing the presence of a not significant effect. The total sample size of the meta-analysis was below the required information size (vertical red line, *n* = 1804 implants), indicating the need for more trials, as the meta-analysis had not sufficient power to detect a better performance of long over short implants.

#### 3.5.2. Peri-Implant Marginal Bone Level Change (MBLc)

Two different analyses were conducted based on the baseline considered in each article for the measurements of MBLc: implant placement or prosthetic loading.

**Figure 5:**
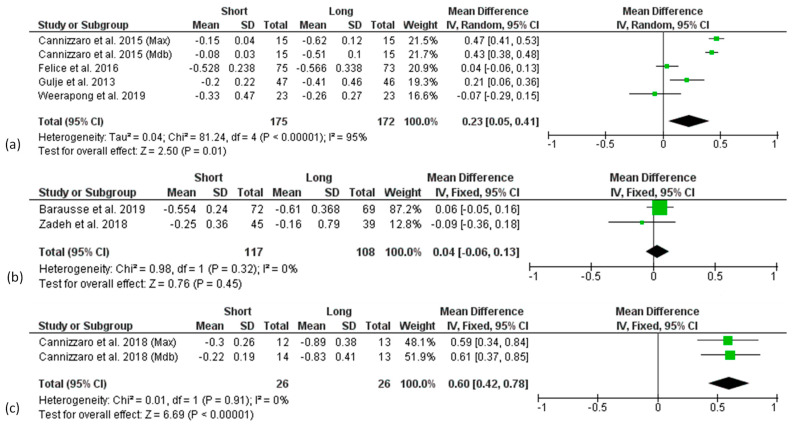
Forest plots reporting difference in means for Marginal Bone Level change (MBLc) between short and long implant groups at 1 (**a**), 3 (**b**), and 5 (**c**) years considering implant placement (IP) as baseline.

Three articles chose implant placement [34,51,52] and six articles chose implant loading [35,45,46,48,49,53] as a baseline for peri-implant MBLc measurements, and they were separately analysed (Figure 4 and Figure 5, respectively); in two studies (three articles) [43,44,50] implants were immediately loaded after placement; thus, they were included in both analyses. Only one article [47] reported values for both implant placement and prosthetic loading as the baseline. The majority of the studies considered MBLc at patient level, only 2 articles reported MBLc at implant level [45,48], while one study [52] reported values both at implant and patient level. In one case, data were both patient and implant level, since one implant per patient was considered [50].

Due to the limited number of the available articles, studies reporting data at implant level were analysed together with studies reporting data at patient level.

A high heterogeneity was found in the 1- and 5-year follow-up analysis considering prosthetic loading as baseline and in the 1-year analysis considering implant placement as baseline, so that a random effect model was used, whereas for all the other analyses, a fixed-effect model was applied.

**Figure 6:**
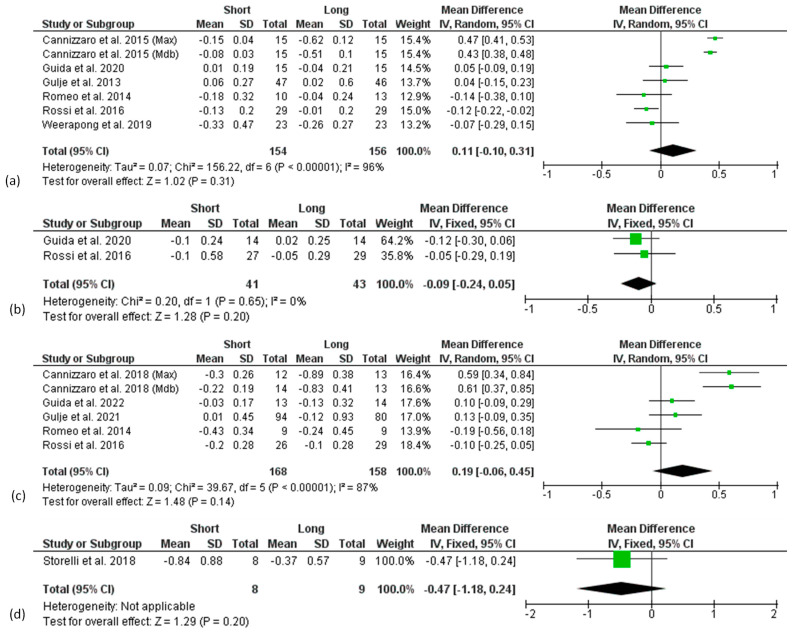
Forest plots reporting difference in means for Marginal Bone Level change (MBLc) between short and long implant groups at 1 (**a**), 3 (**b**), 5 (**c**), and 10 (**d**) years considering prosthetic loading (PL) as baseline.

No difference in the overall effect size was found 3 years after implant placement (Figure 5b) as well as at 1, 3, 5, and 10 years after prosthetic loading (Figure 6a–d).

There was significantly higher marginal bone loss at the 1- and 5-year follow-ups after implant placement for long implants, with a mean difference compared to short implants of 0.23 mm (*p* = 0.01) and 0.60 mm (*p* < 0.00001), respectively (Figure 5a,c), although the analysis was strongly affected by one study [43,44].

#### 3.5.3. Biological and Technical Complications

**Figure 7:**
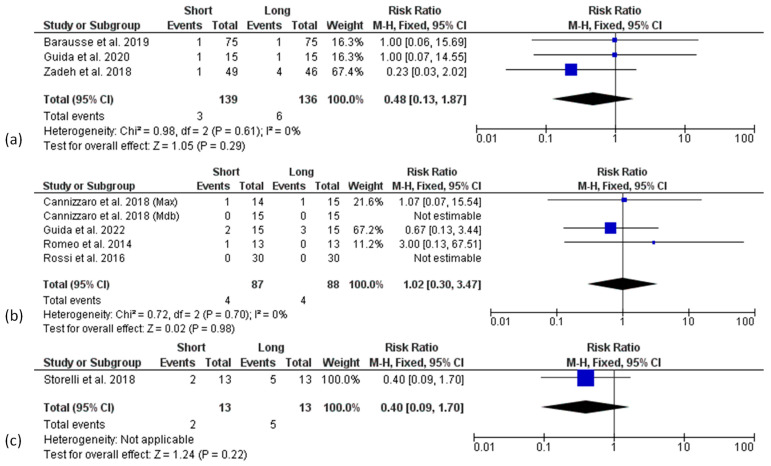
Forest plots (risk ratio, RR) for biological complication rate comparing short and long implant groups at 3 (**a**), 5 (**b**), and 10 years (**c**) of follow-up. RR > 1 indicated a higher complication rate for short than the long implants.

A meta-analysis regarding the biological complications (mucositis and peri-implantitis), and prosthetic complications (implant, prosthesis, abutment or screw fractures, screw loosening, and denture renewal), registered in the included studies was performed. Other reported complications, such as pain, soft tissue ulcerations and wound dehiscence, or loosening of cover/healing screws, were not included among the biological and technical complications, respectively. Data were analysed at the patient level. Three articles provided only implant-level data and were excluded from the analysis of biological [45,48] and technical [45,52] complications. A fixed effect model was used for all the analysis at each follow-up point. None of the 1-year follow-up articles reported patients suffering from mucositis or peri-implantitis. No difference for biological complications between short and long implant groups from 3 to 10 years of follow-up was found (Figure 7a–c).

**Figure 8:**
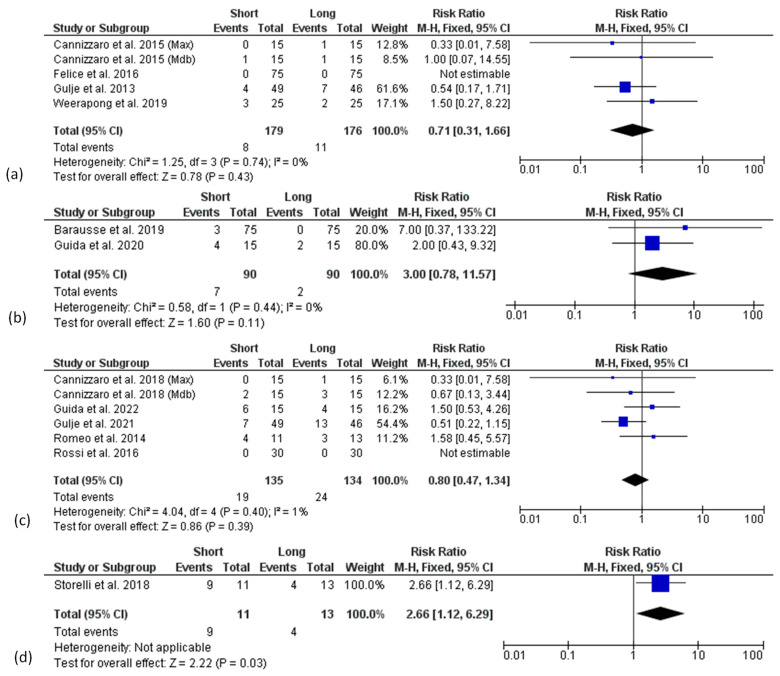
Forest plots (risk ratio, RR) for technical complication rate comparing short and long implant groups at 1- (**a**), 3- (**b**), 5- (**c**) and 10-year follow-up (**d**). RR > 1 indicated a higher complication rate for short rather than long implants.

Similarly, no significant differences in terms of technical complication rates between groups were found at the 1-year, 3-year, and 5-year follow-ups (Figure 8a–c). Only at 10 years was a higher technical complication rate found in the short implant group, although the analysis included only one article [53].

## 4. Discussion

The present meta-analysis exclusively included randomized controlled trials comparing short (≤6 mm) and longer (≥8.5 mm) implants placed in sites without the need for bone augmentation.

Universally accepted definitions of a “short”, “standard”, or “long” implant in the literature does not yet exist, and these concepts continuously evolved throughout the years [27,45,56]. In accordance with the 2018 ITI Consensus Report [12], the authors considered truly “short” implants of length ≤ 6 mm, in comparison to control implants with a length ≥ 8.5 mm.

Short implants have been extensively proposed in atrophic sites as an alternative to longer implants associated with bone augmentation, finding in this treatment their natural term of comparison.

Dental implants placed in vertically augmented bone showed a high survival rate (98%, range 95–100%), comparably to short implants placed in atrophic sites (96%, range 86.7–100%) after periods of 1 to 5 years in function, although they were associated with a higher number of complications, surgical time, and treatment costs [12].

The authors believe, however, that comparison of short and long implants in the same clinical scenario could be the only ones able to correctly evaluate their performance avoiding the effect of confounding factors related to the different characteristics of the edentulous sites, especially those related to the augmentation procedure itself.

Only two other meta-analyses, among those available in the literature, addressed this issue, trying to analyse, by specific sub-analyses, the influence on the reported outcomes of the variable “bone augmentation” among the included trials [8,25], thus representing the main term of comparison for our results. With respect to those meta-analyses, however, the authors were able to include a higher number of more recent RCTs [34,35,44,48,49,50,52,53] with longer follow-up, as well as further additional data directly obtained by the authors themselves.

The first relevant finding of the present meta-analysis is the comparable survival rate found between test and control groups at all the available follow-up times, although the low number of available studies limits the strength of the evidence for this outcome. In fact, the available sample size is smaller than the optimal information size and this affects the rating of evidence by the GRADE methods, indicating a moderate quality, as well as the TSA, which revealed the weak power of the meta-analysis findings. Recent systematic reviews on short implants separately performed additional analyses focused on those RCTs comparing short and longer implants placed in non-augmented bone [8,25]. Ravidà et al. (2019) showed homogeneous results in terms of survival rates between trials with or without bone augmentation procedures, thus suggesting no influence of this variable on this outcome. It must be underlined that just one study of only one follow-up period (one year) was included in the “non-augmentation” group by the authors. Yu et al. (2021) performed a similar sub-group analysis on a larger number of studies and follow-up periods (1, 3, and 5 years). In this case, accordingly with our analysis, a trend for a higher risk of failure for short implants at 1 and 3 years was found, which reached the significance, differently from our results, at 5 years of follow-up with an RR of 0.955 (95% CI 0.912–0.999, *p* < 0.05). Several factors must be considered for a correct interpretation of such different findings, including the type of edentulism, the loading time, and the prosthetic design. Indeed, if we look at the trials reporting higher risk of failure for short implants at 5 years in the study by Yu et al. (2021), the authors find that all the implants were early loaded and supported single crowns or 2/3-units fixed partial prostheses. In contrast, two over the three additional data sets (from two studies [35,44]) included in our analysis regarded splinted implants, conventionally loaded, supporting full-arch rehabilitations of mandibular edentulism. Moreover, details on other important variables (such as bruxism, smoke habit, bone quality and implant stability) in the patients experiencing implant loss were rarely provided by the primary studies, making it more difficult to correctly analyse possible causes and risk factors of implant failures.

Peri-implant marginal bone level maintenance over time is of pivotal importance for the long-term success of dental implants. The present meta-analysis showed that only 1- and 5-year follow-up data from implant placement showed a significantly higher bone loss for long implant group. However, such results must be considered with caution due to the very low number of included studies and the high heterogeneity found, with a significant impact on the overall evaluation given by two articles [43,44] derived from the same study. This latter result was the only one included in the “no augmentation” group by Ravidà et al. [8] in their meta-analysis, resulting in a significantly higher bone loss for the long implant group at the 1-year follow-up. Also, Yu et al. [25] performed a sub-group analysis for MBLc on “augmentation” vs. “no augmentation” studies one year after two baseline points (implant placement and prosthetic loading). They did not find a difference between long and short implants both one year after prosthetic loading and, different from the study results, also one year after implant placement. Also in this case, the low number of included studies and their heterogeneity impose caution.

In addition, the incidence of the reported biological and technical complications did not show a statistically significant difference between short and long implants, in line with the analyses performed by previous similar reviews up to 5 years of follow-up [8,25]. At longer follow-ups, a statistically significant higher number of technical complications was found after 10 years [53], although the value of this finding was sustained by only one trial. It is hard, however, to compare the results with those from other similar studies, in light of the different biological complications considered (only the occurrence of peri-implant mucositis and peri-implantitis was analysed in our study), and, moreover, of the different diagnostic criteria adopted for such diseases among the studies.

Looking at the main limitations of the present meta-analysis, the low number of includible studies and their mainly short/medium follow-ups must be cited. Furthermore, the variety of experimental conditions among studies limit the power of the obtained results, and no further analyses could be performed based on variables including implant location (mandible vs. maxilla), type of edentulism (total vs. partial), implant type, smoke habit, implant loading, periodontal health, systemic conditions due to the reduced number of available studies, or the lack of information retrievable about these variables. No subgroup analysis could be performed for confounding factors, such as smoking and periodontal status, or for the level of analysis (implant or patient level). Implant-level and patient-level data, were pooled together in the MBLc meta-analysis, with a consequent possible underestimation of the confidence intervals for the pooled estimate.

Further, well-designed RCTs comparing clinical and radiological outcomes of short and long implants placed in similar conditions (location, type of edentulism, prosthetic rehabilitation, etc.) and with an adequate analysis of confounding factors should be performed to obtain more solid evidence about the efficacy of short implants used in atrophic sites.

This approach would also help to extend their clinical indications, supporting the hypothesis of their routine use in non-atrophic edentulous sites instead of traditionally long implants, in the groove of a minimally invasive, low-stress, simplified implant therapy, with benefits for both patients and clinicians.

The ideal length of implants supporting prosthetic rehabilitations, indeed, is a relative concept that underwent a progressive reduction throughout the years from the origin of modern implantology to the present [27,45,56], also thanks to the outstanding progress made in the last decades in terms of constitutive materials and macro-design of dental implants, as well as of micro/nano-topographic and chemical modifications of modern implant surfaces [57,58].

The advantages of using short implants in non-atrophic sites compared to longer ones include a simplified surgical management during placement, with a lower risk of involving noble anatomic structures, as well as a simplified and less invasive surgical procedure, if biological complications, over time, might require fixture removal, with the chance to leave an amount of residual bone enough for a new rehabilitation. On the other hand, due to the reduced fixture length, some conditions, such as low-density bone and operative protocols, e.g., post-extraction placement, and immediate/early loading, could reduce the reliability and predictability of this rehabilitative approach.

This is the reason, in the authors’ opinion, because further RCTs should be performed in which all the possible relevant variables and risks are considered, analysed, and controlled in order to obtain more solid evidence on the performance of short implants in each specific clinical scenario, starting from the “low-risk” one (healed sites, good bone quality, conventional loading, splinted implants), to the others.

## 5. Conclusions

Moderate evidence exists suggesting that short implants perform, in the medium term, as well as longer ones in the rehabilitation of edentulous sites without the need for bone augmentation. However, long-term data are lacking and the experimental conditions among the analysed studies are heterogeneous. Further data from long-term, well-designed RCTs conducted under comparable clinical conditions, in terms of location and type of edentulism, prosthetic design, surgical and loading timing, and with a proper analysis of potential confounding factors, are needed. In this way it will be possible to provide specific, evidence-based, clinical recommendations and reliable indications for an extended use of short implants, also in the case of non-atrophic sites.

## Figures and Tables

**Table 1:** Detailed search strategy for electronic databases.

Database	Search String
PubMed (MEDLINE)	(“dental implants” [MeSH Terms] OR “dental implantation” [MeSH Terms]) AND (short OR shorter OR long OR longer OR length) AND (randomized controlled trials)
Clinicaltrials.gov	Condition or disease: dental implants OR dental implantation Other terms: short OR shorter OR long OR longer OR length
Embase	(‘dental implants’/exp OR ‘dental implants’ OR ‘dental implantation’/exp OR ‘dental implantation’) AND (short OR shorter OR long OR longer OR ‘length’/exp OR length) AND randomized AND controlled AND trial
Scopus	(‘dental AND implants’/exp OR ‘dental AND implants’ OR ‘dental AND implantation’/exp OR ‘dental AND implantation’) AND (short OR shorter OR long OR longer OR ‘length’/exp OR length) AND (randomized AND controlled AND trial)
Open grey (assimilated by Dans Easy)	(“dental implants” OR “dental implantation”) AND (short OR shorter OR long OR longer OR length) AND (randomized controlled trials)

**Table 2:** Excluded studies and reasons for exclusion.

Study	Reason for Exclusion
Sahrmann et al., 2016 [36]	Sinus floor elevation with Summer’s technique allowed (reason 1)
Naenni et al., 2018 [37]	Sinus floor elevation with Summer’s technique allowed (reason 1)
Sahrmann et al., 2017 [38]	Only rx bone density analysis (reason 2)
Sluka et al., 2020 [39]	Only rx bone radiopacity analysis (reason 2)
Della Vecchia et al., 2018 [40]	Mini implants for overdentures (reason 3)
Esposito et al., 2015 [41]	Follow up <12 months (reason 4)
Al-Hashedi et al., 2016 [42]	<8.5 mm implants in the control group (reason 5)

**Table 3:** Characteristics of the included studies.

Study/Studies	Study Design	Follow Up (Years)	Max, Mdb	Test Implant Length	Control Implant Length	Patients ^1^/Implants		Age (Mean) Age (Range) Gender (M/F)		Implant Surface (Name, Company)	Implant Location	Prosthetic Rehabilitation	Prosthetic Loading	Post-Extraction Implants Included
						Test	Control	Test	Control					
Gulje et al., 2013 [47], Zadeh et al., 2018 [52]; Gulje et al., 2021 [48]	RCT, parallel group	1, 3, 5	Max + Mdb pooled	6 mm	11 mm	49/108	46/101	54.8 26–69 21/28	54.1 34–70 27/19	Blasted fluoride-modified (OsseoSpeed, Astra Tech Implant System, Dentsply Sirona)	Premolar and molar	2–3 unit splinted crowns (screw-retained)	Early (6 w)	No
Romeo et al., 2014 [46]; Storelli et al., 2018 [53]	RCT, parallel group	5, 10	Max + Mdb pooled	6 mm	10 mm	11/26	13/28	50 37–75 6/5	56 32–75 6/7	Sand blasted large grit acid etched (SLA, Straumann)	Premolar and molar	2–3 unit splinted crowns (cemented)	Early (8 w)	No
Cannizzaro et al., 2015 [43]; 2018 [44]	RCT, parallel group	1, 5	Max, Mdb	5 mm	11.5 mm	Max 15/91 Mdb 15/60	Max 15/90 Mdb 15/62	Max 58.9 44–78 7/8 Mdb 62.9 47–80 8/7	Max 58.5 43–72 9/6 Mdb 58.8 38–72 7/8	Dual acid-etched (NanoTite, Biomet 3I)	Anterior and posterior	FA prostheses with distal cantilever (screw-retained)	Immediate (<1 w)	Yes
Rossi et al., 2016 [45]	RCT, mixed ^2^	5	Max + Mdb pooled	6 mm	10 mm	NC/30	NC/30	48.8 NR 16/14	47.7 NR 16/14	Sand blasted large grit acid etched (SLA, Straumann)	Premolar and molar	SCs (retention NC)	Early (7 w)	No
Felice et al., 2016 [51]; Barausse et al., 2019 [34]	RCT, parallel group	1, 3	Max + Mdb pooled	4 mm	≥8.5 mm	75/124	75/116	53.7 27–76 30/45	55.5 25–86 36/39	Sand-blasted acid-etched (SA_2_, TwinKon, Global D)	Premolar and molar	SCs and 2–3 unit splinted crowns (screw retained)	Conventional (4 m)	Yes
Weerapong et al., 2019 [50]	RCT, parallel group	1	Mdb	6 mm	10 mm	25/25	25/25	50.5 20–61 9/14	51.4 22–64 7/16	NR (PW+ Dental Implant System)	Molar	SCs (cemented)	Immediate (<1 w)	Yes
Guida et al., 2020 [49]; Guida et al., 2022 [35]	RCT, parallel group	1, 3, 5	Mdb	6 mm	11 mm	15/75	15/75	63 NR 5/10	61 NR 12/3	Blasted fluoride-modified (OsseoSpeed, Astra Tech Implant System, Dentsply Sirona)	Interforaminal	FA prostheses with distal cantilever (screw-retained)	Conventional (3 m)	No

^1^ Number of randomized patients. ^2^ Patients with both test and control sites were permitted. RCT: randomized controlled trial; Max: maxilla; Mdb: mandible; FA: full-arch; SCs: single crowns; NR: not reported; NC: not clear.

**Table 4:** Grades of recommendation, assessment, development, and evaluation (GRADE) approach summarizing the evidence. Question: Short implants (≤6 mm) compared to longer implants (≥8.5 mm) in edentulous sites without the need for bone augmentation. Moderate certainty: The authors are moderately confident in the effect estimate; the true effect is likely to be close to the estimate of the effect, but there is a possibility that it is substantially different. Generated by GRADEpro GDT web application, http://gradepro.org, (accessed on 20 February 2020). CI: confidence interval; RR: risk ratio; MD: mean difference. ^a^ Small simple size (less than optimal information size), CI of RR included 1.

Certainty Assessment							№ of Implants		Effect		Certainty	Importance
№ of Studies	Study Design	Risk of Bias	Inconsistency	Indirectness	Imprecision	Other Considerations	Short Implants	Long Implants	Relative (95% CI)	Absolute (95% CI)		
Survival Rate (Implant Level) (Follow-Up: Range 1 to 10 Years)												
8	Randomised trials	Not serious	Not serious	Not serious	Serious ^a^	None	518/540 (95.9%)	515/526 (97.9%)	RR 0.98 (0.96 to 1.00)	20 fewer per 1.000 (from 39 fewer to 0 fewer)	⊕⊕⊕◯ Moderate	CRITICAL

## Data Availability

Not applicable.
